# Modeling the Non-Equilibrium Process of the Chemical Adsorption of Ammonia on GaN(0001) Reconstructed Surfaces Based on Steepest-Entropy-Ascent Quantum Thermodynamics

**DOI:** 10.3390/ma10080948

**Published:** 2017-08-15

**Authors:** Akira Kusaba, Guanchen Li, Michael R. von Spakovsky, Yoshihiro Kangawa, Koichi Kakimoto

**Affiliations:** 1 Department of Aeronautics and Astronautics, Kyushu University, Fukuoka 819-0395, Japan; kangawa@riam.kyushu-u.ac.jp (Y.K.); kakimoto@riam.kyushu-u.ac.jp (K.K.); 2 Department of Engineering Science, University of Oxford, Parks Road, Oxford OX1 3PJ, UK; guanchen.li@eng.ox.ac.uk; 3 Center for Energy Systems Research (CESR), Mechanical Engineering Department, Virginia Tech, Blacksburg, VA 24061, USA; vonspako@vt.edu; 4 Research Institute for Applied Mechanics (RIAM), Kyushu University, Fukuoka 816-8580, Japan; 5 Center for Integrated Research of Future Electronics (CIRFE), Institute of Materials and Systems for Sustainability (IMaSS), Nagoya University, Nagoya 464-8601, Japan

**Keywords:** metalorganic vapor phase epitaxy, gallium nitride, chemical adsorption, surface reconstruction, density functional theory calculations, steepest-entropy-ascent quantum thermodynamics

## Abstract

Clearly understanding elementary growth processes that depend on surface reconstruction is essential to controlling vapor-phase epitaxy more precisely. In this study, ammonia chemical adsorption on GaN(0001) reconstructed surfaces under metalorganic vapor phase epitaxy (MOVPE) conditions (3Ga-H and N_ad_-H + Ga-H on a 2 × 2 unit cell) is investigated using steepest-entropy-ascent quantum thermodynamics (SEAQT). SEAQT is a thermodynamic-ensemble based, first-principles framework that can predict the behavior of non-equilibrium processes, even those far from equilibrium where the state evolution is a combination of reversible and irreversible dynamics. SEAQT is an ideal choice to handle this problem on a first-principles basis since the chemical adsorption process starts from a highly non-equilibrium state. A result of the analysis shows that the probability of adsorption on 3Ga-H is significantly higher than that on N_ad_-H + Ga-H. Additionally, the growth temperature dependence of these adsorption probabilities and the temperature increase due to the heat of reaction is determined. The non-equilibrium thermodynamic modeling applied can lead to better control of the MOVPE process through the selection of preferable reconstructed surfaces. The modeling also demonstrates the efficacy of DFT-SEAQT coupling for determining detailed non-equilibrium process characteristics with a much smaller computational burden than would be entailed with mechanics-based, microscopic-mesoscopic approaches.

## 1. Introduction

GaN and related alloys are well-known materials for UV/blue light-emitting diodes (LEDs) and laser diodes (LDs) [1,2,3]. High quality AlGaN and InGaN growth with various orientations have been actively studied with the aim of producing high-efficiency emissions and extending the emission wavelength [4,5,6]. These days, GaN is attracting much attention as a material for the next generation of high power devices [7,8]. To realize device-grade crystals for this purpose, the metalorganic vapor phase epitaxy (MOVPE) process must be controlled more precisely and high quality substrates using liquid-phase growth methods [9,10] are needed. Thus, it is essential to clearly understand the elementary growth processes involved in MOVPE such as adsorption-desorption.

It is well known that reconstructed structures on growth surfaces depend on temperature and ambient partial pressures or beam equivalent pressures (BEP) [11]. Of course the behavior of elementary growth processes varies with reconstructed surfaces. Unlike the case of molecular beam epitaxy (MBE), the in-situ observation of reconstructed structures during MOVPE is difficult because electron diffraction methods such as RHEED are not available at non-vacuum MOVPE pressures. Thus, theoretical predictions based on first-principle calculations and statistical mechanics [12,13,14] are very important. Some groups have reported the prediction of GaN surface structures for the MOVPE process [15,16,17,18,19,20]. 3Ga-H, and N_ad_-H + Ga-H structures can appear in (0001) at ordinary conditions according to the literature [20].

However, the first-principle predictions made in the literature using, for example, density functional theory (DFT) are equilibrium based and unable to capture the non-equilibrium kinetic characteristics of the process. To do so, DFT can be coupled to steepest-entropy-ascent quantum thermodynamics (SEAQT), which is a thermodynamic-ensemble based, first-principles framework that can predict the behavior of non-equilibrium processes even those far from equilibrium. This framework has been developed and applied to both non-reacting and reacting systems at multiple spatial and temporal scales and validated via comparisons with experiments [21,22,23,24,25,26,27,28,29,30,31,32,33,34,35,36,37,38,39,40,41]. It complements the postulates of quantum mechanics (QM) with the second law of thermodynamics and provides an equation of motion, which includes the linear unitary dynamics of QM as a special case and a non-linear dynamics, which captures the irreversible relaxation of system state. A density of states method developed by Li and von Spakovsky [31] extends the computational applicability of this framework to infinite-dimensional state spaces and as a consequence to all spatial and temporal scales. Furthermore, introduction of the concept of hypoequilibrium state by Li and von Spakovsky [31] leads to a generalization of the thermodynamic description even into the far-from-equilibrium realm [32], leading to a very useful tool for modeling a variety of practical engineering problems at multiple scales. An example of this is the use of SEAQT to model multiple oxygen and chromium oxide reduction pathways in a solid oxide fuel cell (SOFC) cathode, where coupled mass and heat diffusion and electrochemical and chemical reactions are treated in a single framework across multiple spatial and temporal scales, providing guidance for cathode design [37,38]. For other practical applications of this framework, the reader is referred to [30,31,32,33,34,35,36,39].

In the present study, the kinetics of chemical adsorption on a semiconductor surface is modeled using SEAQT and the energy eigenstructure generated by DFT. Since, in general, the chemical adsorption process begins from a highly non-equilibrium state, SEAQT is an ideal choice for handling this problem on a first-principles basis. The results of this analysis are the dimensionless time evolutions of adsorption probabilities (i.e., the chemical kinetics) and of system temperature. These results are much more informative than the energetics of DFT alone and are helpful in clearly understanding the elementary growth processes involved. In what follows, Section 2 provides a brief description of the underlying theory and non-equilibrium model developed, while Section 3 presents and discusses a number of the results generated. Section 4 concludes with a set of pertinent remarks.

## 2. Theory and Model

### 2.1. SEAQT Equation of Motion

In this subsection, the theory of SEAQT for dilute-Boltzmann-gas is briefly introduced. For a complete and general discussion, the reader is referred to [21,22,23,24,25,28,30,31,32,33,34,36,40,41]. In a quantum system, energy takes discrete values (i.e., energy eigenlevels). The thermodynamic state of the system is defined via a probability distribution $\left\{p_{i}\right\}$ among the energy eigenlevels $\left\{\epsilon_{i}\right\}$, which may have degeneracy $\left\{n_{i}\right\}$. A thermodynamic property of the system is defined as the ensemble average,

(1) $$E=\langle e\rangle =\underset{i}{\sum }p_{i}\epsilon_{i},$$

(2) $$S=\langle s\rangle =\underset{i}{\sum }-p_{i}\ln\left(\frac{p_{i}}{n_{i}}\right).$$

Here, the von Neumann expression for entropy is used [42,43]. An equation of motion for $p=\left\{p_{i}\right\}$ describing system state evolution is expressed in general terms as [31]

(3) $$\frac{dp}{dt}=X_{p\left(t\right)}+Y_{p\left(t\right)},$$

where the reversible dynamics is represented by $X_{p\left(t\right)}$ and the irreversible relaxation by $Y_{p\left(t\right)}$. The reversible dynamics follows Liouville’s equation or the Schrödinger equation and the irreversible dynamics is related to the entropy generation, which in the SEAQT framework is determined via the principle of steepest entropy ascent. For a dilute-Boltzmann-gas, the reversible part vanishes and the system follows a dynamics driven by entropy generation only. This is a good assumption for the present work, since the timescale of the reversible dynamics (i.e., of the bulk flow) is orders of magnitude slower than that of the irreversible dynamics of the chemical kinetics. Thus, it is reasonable to assume that the two dynamics are decoupled. Since the focus in this paper is only on the chemical kinetics, i.e., the irreversible part of the equation of motion, the reversible part vanishes.

According to the principle of SEA, the irreversible relaxation is in the direction that has the largest entropy gradient consistent with the conservation laws for mass and energy, i.e., in this case, the total probability and total energy remain constant. Using the mathematical representation of the steepest-entropy-ascent direction [23], the SEA equation of motion takes the following form:

(4) $$\frac{dp_{i}}{dt}=Y_{p\left(t\right),i}=\frac{1}{\tau }\frac{\left|\begin{array}{ccc} -p_{i}\ln\left(\frac{p_{i}}{n_{i}}\right) & p_{i} & p_{i}\epsilon_{i}\\ \langle s\rangle & 1 & \langle e\rangle \\ \langle es\rangle & \langle e\rangle & \langle e^{2}\rangle \end{array}\right|}{\left|\begin{array}{cc} 1 & \langle e\rangle \\ \langle e\rangle & \langle e^{2}\rangle \end{array}\right|},$$

where τ is the relaxation time and the ensemble averages appearing in this equation are given by

(5) $$\langle es\rangle =\underset{i}{\sum }-p_{i}\epsilon_{i}\ln\left(\frac{p_{i}}{n_{i}}\right),$$

(6) $$\langle e^{2}\rangle =\underset{i}{\sum }p_{i}\epsilon_{i}^{2}.$$

Note that even though the absolute value of the relaxation time τ influences the dynamics of the state evolution, i.e., the speed at which the system moves along the kinetic path predicted by Equation (4), it does not impact the kinetics itself. For a proof, see [31]. Thus, the use of a dimensionless time as is done here does not corrupt the first-principle nature of the kinetic results of state evolution presented since this dimensionless time is not empirically but fundamentally defined based on the SEA principle. Furthermore, to study the dynamics of the kinetic path, an absolute value for τ could be determined *ab initio* based on quantum mechanics and state space geometric considerations as outlined in [41]. However, it is not necessary for the present study and, thus, beyond the scope of this paper.

As to a rigorous derivation of Equation (4), it is based on a number of state space geometric considerations (e.g., the geometry of a manifold) relative to the principle of steepest entropy ascent or equivalently maximum entropy production. For details, the reader is referred to [21,22,23,24,25,31]. The mathematical expression of the steepest-entropy-ascent direction is given by the perpendicular component of the entropy gradient to the manifold in state space (e.g., Hilbert space) spanned by the total energy and probability gradients. The dissipation term of Equation (4) is then constructed as a ratio of Gram determinants based on these gradients expressed in terms of the thermodynamic properties seen in the equation above. As proven in [21], Equation (4) inherently satisfies the postulates of quantum mechanics as well as the first and second laws of thermodynamics, and the path predicted by this equation is the unique thermodynamic path along which the state of the system evolves in time.

Finally, once the energy eigenstructure of the system (i.e., $\left\{\epsilon_{i}\right\}$ and $\left\{n_{i}\right\}$) is known, the state evolution from any initial non-equilibrium state to stable equilibrium is determined via the system of equations formed by Equation (4). The eigenstructure for the system considered here is presented in the following section.

### 2.2. System and Energy Eigenstructure

In the present study, chemical adsorption of ammonia on 3Ga-H and N_ad_-H + Ga-H structures is modeled. The corresponding reaction mechanisms are

NH_3_(g) + S[3Ga-H] → H_2_(g) + S[NH_2_(br) + 2Ga-H], (7)

NH_3_(g) + S[N_ad_-H + Ga-H] → H_2_(g) + S[N_ad_-H + Ga-NH_2_]. (8)

**Figure 1:**
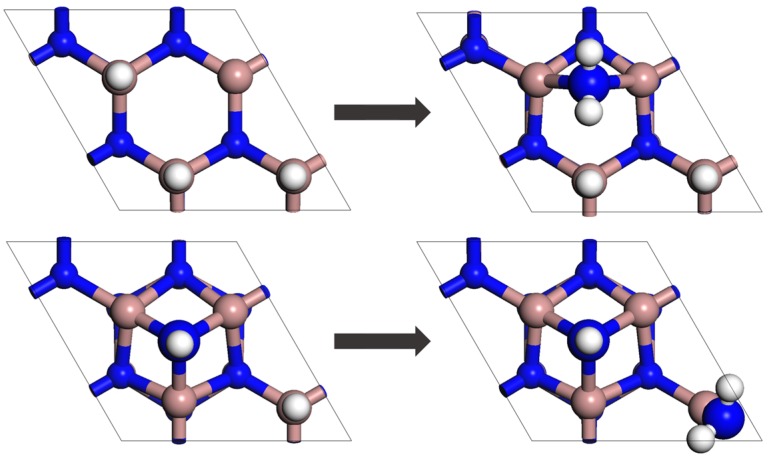
Surface structures before and after the chemical adsorption reactions: (upper row) NH_3_(g) + S[3Ga-H] → H_2_(g) + S[NH_2_(br) + 2Ga-H], (lower row) NH_3_(g) + S[N_ad_-H + Ga-H] → H_2_(g) + S[N_ad_-H + Ga-NH_2_]. Brown, blue, and white atoms are gallium, nitrogen, and hydrogen, respectively.

The surface structures before and after these reactions are shown in Figure 1. The energy eigenstructure for each of these chemically reactive systems is decomposed into two subsystem eigenstructures, one for the reactants and the other for the products. For the system subject to reaction mechanism (7), subsystem 1 (i.e., the reactants) is comprised of one NH_3_ molecule and the 2 × 2 surface S[3Ga-H], while subsystem 2 (i.e., the products) is comprised of one H_2_ molecule and the 2 × 2 surface S[NH_2_(br) + 2Ga-H]. In a like manner, for the system subject to reaction mechanism (8), subsystem 1 (i.e., the reactants) is comprised of one NH_3_ molecule and the 2 × 2 surface S[N_ad_-H + Ga-H], while subsystem 2 (i.e., the products) is comprised of one H_2_ molecule and the 2 × 2 surface S[N_ad_-H + Ga-NH_2_]. The energy eigenlevels of the eigenstructures for subsystems 1 and 2 are then given by

(9) $$\epsilon_{i}^{\mathrm{sub}1}=E_{\mathrm{DFT}}^{\mathrm{sub}1}+E_{\mathrm{ZPV}}^{\mathrm{NH}3}+E_{\mathrm{ZPV}}^{\mathrm{ad}1}+\epsilon_{i}^{\mathrm{NH}3},$$

(10) $$\epsilon_{i}^{\mathrm{sub}2}=E_{\mathrm{DFT}}^{\mathrm{sub}2}+E_{\mathrm{ZPV}}^{H2}+E_{\mathrm{ZPV}}^{\mathrm{ad}2}+\epsilon_{i}^{H2},$$

where *i* is the index of the energy eigenlevel; $\left\{\epsilon_{i}^{\mathrm{sub}1}\right\}$ and $\left\{\epsilon_{i}^{\mathrm{sub}2}\right\}$ are the energy eigenlevels of subsystems 1 and 2; $E_{\mathrm{DFT}}^{\mathrm{sub}1}$ and $E_{\mathrm{DFT}}^{\mathrm{sub}2}$ are the total energies of these subsystems determined using DFT; $E_{\mathrm{ZPV}}^{\mathrm{NH}3}$ and $E_{\mathrm{ZPV}}^{H2}$ are the zero-point energies of the NH_3_ and H_2_ molecules, respectively; $E_{\mathrm{ZPV}}^{\mathrm{ad}1}$ and $E_{\mathrm{ZPV}}^{\mathrm{ad}2}$ are the zero-point energies of the subsystem adsorbates calculated from the vibrational frequencies of the adsorbates. The $\left\{\epsilon_{i}^{\mathrm{NH}3}\right\}$ and $\left\{\epsilon_{i}^{H2}\right\}$ are the energy eigenlevels of the NH_3_ and H_2_ molecules, respectively, and are constructed from the energy eigenlevels of each degree of freedom of the molecules, i.e., translation, rotation and vibration, which are determined using the infinite potential well, the rigid motor, and the harmonic oscillator models, i.e.,

(11) $$D_{\mathrm{tra}}\left(\epsilon_{\mathrm{tra}}\right)=\frac{2\pi V}{h^{3}}{\left(2m\right)}^{\frac{3}{2}}{\epsilon_{\mathrm{tra}}}^{\frac{1}{2}},$$

(12) $$D_{\mathrm{rot}}^{\mathrm{linear}}\left(\epsilon_{\mathrm{rot}}\right)=\frac{1}{\sigma B},\;B=\frac{h^{2}}{8\pi^{2}I_{B}},$$

(13) $$D_{\mathrm{rot}}^{\mathrm{non-linear}}\left(\epsilon_{\mathrm{rot}}\right)=\frac{2}{\sigma {\left(B_{\mathrm{av}}\right)}^{\frac{3}{2}}}{\epsilon_{\mathrm{rot}}}^{\frac{1}{2}},\;B_{\mathrm{av}}={\left(ABC\right)}^{\frac{1}{3}},$$

(14) $$\epsilon_{\mathrm{vib}}=nh\nu ,\;n=0,\;1,\;2,\;\cdots .$$

The translational and rotational energy eigenlevels $\epsilon_{\mathrm{tra}}$, $\epsilon_{\mathrm{rot}}$ are treated as quasicontinuous [31] and the associated energy eigenstructures are presented using the density of states $D_{\mathrm{tra}}$ and $D_{\mathrm{rot}}$, since the characteristic temperatures of translation and rotation are much smaller than the temperatures studied. In Equation (11), V is the volume, h is Planck’s constant, and m is the particle mass. Equations (12) and (13) are the rotational density of states for the linear molecules (i.e., H_2_) and the non-linear molecules (i.e., NH_3_), respectively. I in these equations is the moment of inertia, while A, B, C are the rotational constants, $B_{\mathrm{av}}$ is the geometrical mean of the rotational constants, and σ is the symmetry factor. When A=B=C (i.e., $B_{\mathrm{av}}=B$), Equation (13) corresponds to the expression for a spherical top. The use of this expression with $B_{\mathrm{av}}$ for the NH_3_ molecule is an approximation. In Equation (14), the $\epsilon_{\mathrm{vib}}$ are the discrete eigenenergies for vibrational motion, n is the quantum number, and ν is the vibrational frequency. The procedure for developing each subsystem energy eigenstructure using Equations (11) and (12) can be found in Reference [31]. In a similar way, that for the non-linear molecules is developed. The final energy eigenstructure for each reactive system is then given by $\left\{\epsilon_{i}\right\}=\{\epsilon_{i}^{\mathrm{sub}1},\epsilon_{i}^{\mathrm{sub}2}$}. In order to closely approximate the system’s non-equilibrium state evolution in infinite-dimensional state space with an effective finite-dimensional one, the SEAQT equation of motion, Equation (4), is numerically solved using the density of states method developed by Li and von Spakovsky [31].

With regard to the DFT calculations, all electron calculations are made using the DMol^3^ software package [44,45] with the Perdew-Burke-Ernzerhof (PBE) functional [46] and the double numerical plus polarization (DNP) basis set for the isolated molecule and the 2 × 2 surface slab model. The slab model comprises a vacuum layer of more than 20 Å and five GaN bilayers whose bottom layer is fixed and passivated with fictitious hydrogen atoms [47]. A basis set cutoff of 4.8 Å and a 3 × 3 × 1 Monkhorst-Pack (MP) *k*-point mesh [48] are used. The geometry optimization convergence thresholds are 2.0 × 10^−5^ Ha, 0.0005 Ha/Å, and 0.005 Å for the energy change, maximum force, and maximum displacement, respectively. For the frequency of the adsorbates, partial Hessian calculations are performed.

### 2.3. Initial State and Model Parameters

In this research, the initial state of the system is chosen to be a second-order hypoequilibrium state [31] for which the probability distribution in each subsystem $\left\{p_{i}^{\mathrm{sub}}\right\}$ takes a canonical form, namely,

(15) $$p_{i}^{\mathrm{sub}}=P^{\mathrm{sub}}\frac{n_{i}^{\mathrm{sub}}\exp\left(-\epsilon_{i}^{\mathrm{sub}}/k_{b}T^{\mathrm{sub}}\right)}{Z^{\mathrm{sub}}}.$$

Here, $T^{\mathrm{sub}}$ represents the subsystem temperature and is set to 1000 °C for both subsystems, while the total probability of subsystem 1, $P^{\mathrm{sub}1}$, is set at 0.99999 and that for subsystem 2 at 0.00001. As is shown in [31], the total probability evolution from a more general initial state (e.g., that of a gamma distribution) is very similar to that of an initial hypoequilibrium state except in the very early stages of the evolution. Thus, using an initial hypoequilibrium state, as is done here, is a good approximation to a very wide range of initial conditions. As to the system volume, it is set equal to 0.001 m^3^. The relaxation time τ in the equation of motion is fixed at 1 so that the unique state evolution predicted for a given initial state describes the kinetics of the state trajectory only and not its dynamics, i.e., the real time required to traverse the trajectory of intermediate non-equilibrium states through which the system passes. To capture the latter, τ can be determined via experiment [27,28,29] or a microscopic/mesoscopic model (e.g., one from kinetic theory) [28,29,32,33,38,39] or in a completely *ab initio* fashion as is done in [41].

## 3. Results and Discussion

### 3.1. Probability Distribution Among Energy Eigenlevels

**Figure 2:**
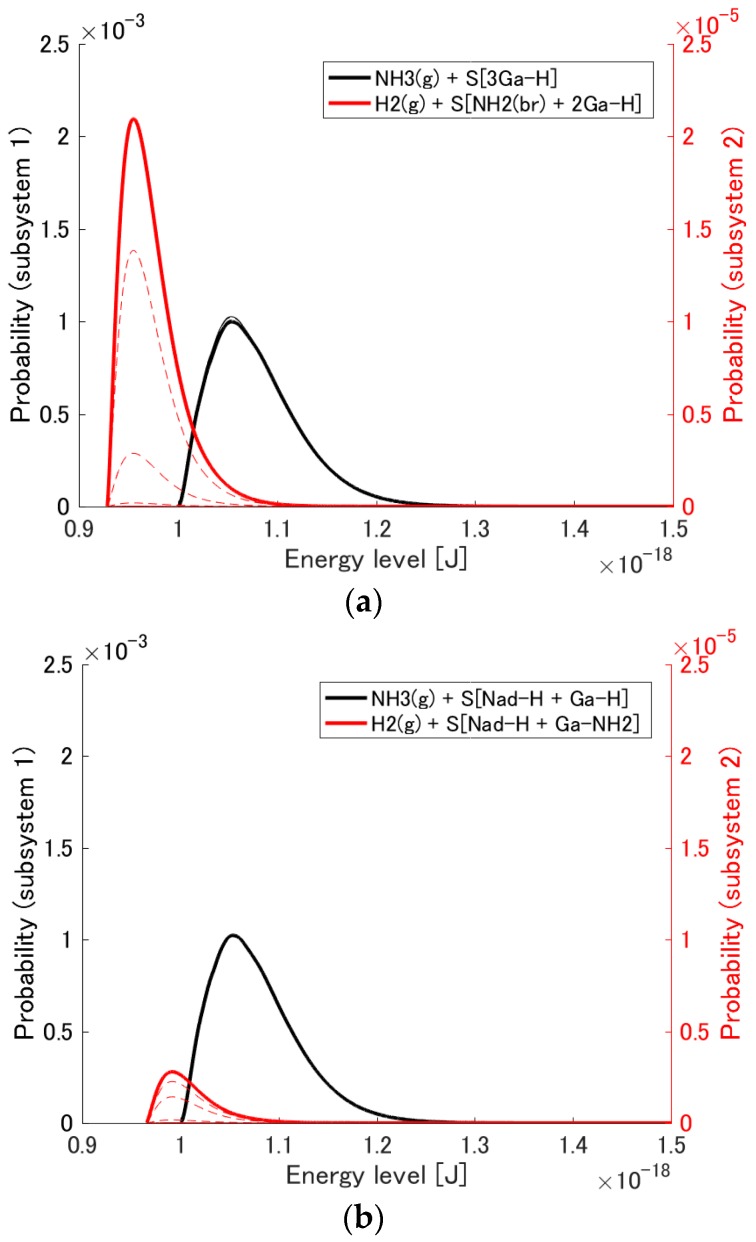

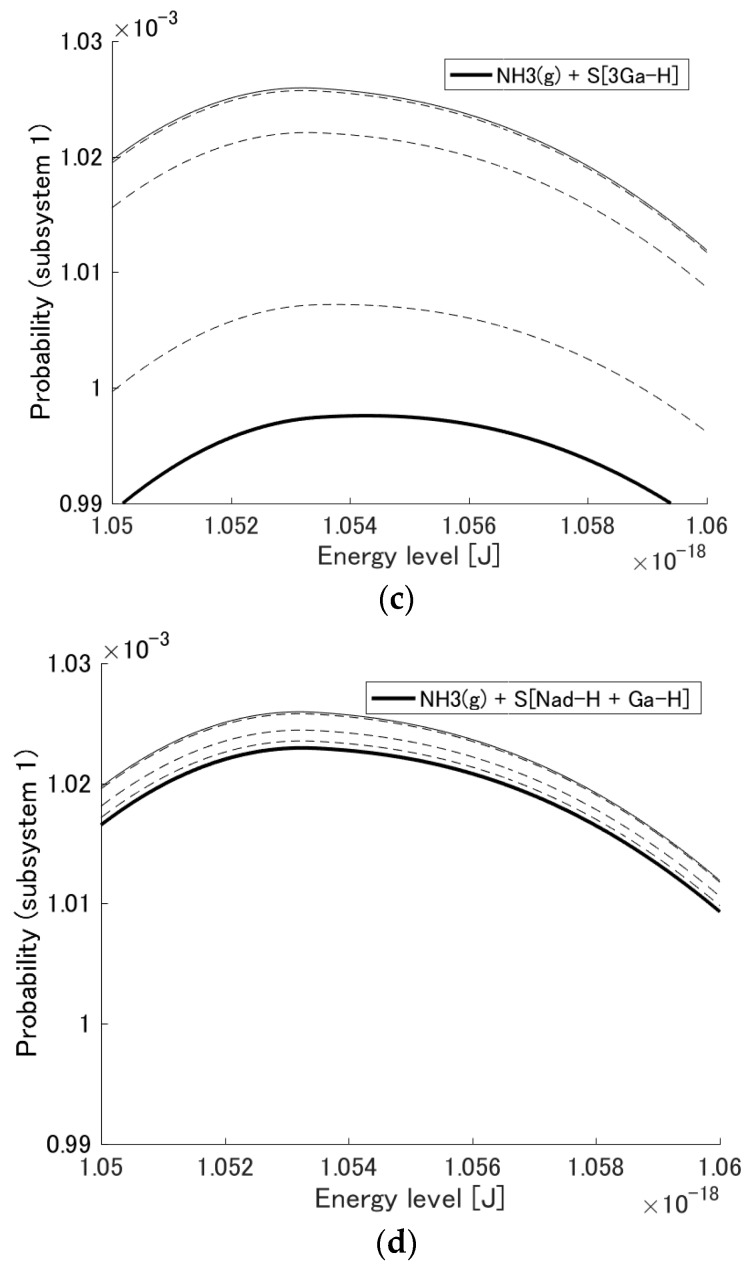
Probability distribution among the energy eigenlevels for the adsorption reactions on (**a**) S[3Ga-H] (and (**c**) zoomed-in) and (**b**) S[N_ad_-H + Ga-H] (and (**d**) zoomed-in). The narrow solid, dashed, and bold solid lines correspond to the initial state, a number of intermediate states during relaxation, and the stable equilibrium state, respectively.

The probability distribution $\left\{p_{i}\right\}$ among the energy eigenlevels $\left\{\epsilon_{i}\right\}$ for the initial state, a number of intermediate states during the relaxation, and the final stable equilibrium state are shown in Figure 2a,b for the systems corresponding to the reaction mechanisms of Equations (7) and (8), respectively. The black curves are the distributions for subsystem 1 (i.e., the reactants), while red ones are those for subsystem 2 (i.e., the products). Note that the vertical axis for subsystem 2 is smaller than that for subsystem 1 by two orders of magnitude. The narrow solid, dashed, and bold solid curves are, respectively, the distributions for the initial state, the intermediate states during the relaxation, and the equilibrium state. The red narrow solid line, which corresponds to the initial state of subsystem 2, essentially lies on the horizontal axis since the probability of finding any products in the system is extremely small. At the initial state and during the relaxation, each state exhibits a canonical distribution among the energy eigenlevels of each subsystem because, as is proven in [31], if the system initially is in a hypoequilibrium state, all intermediate states will also be in hypoequilibrium. At stable equilibrium, the canonical distribution for the whole system is achieved.

As can be seen in the figure, the probability of subsystem 2 for each system (Figure 2a,b) increases as the state evolves. At the same time, that of subsystem 1 for each system (Figure 2a,b) decreases slightly, although without the change of scale seen in Figure 2c,d, this decrease is difficult to observe. Thus, the probability flows from subsystem 1 to subsystem 2 for each system as the chemical adsorption of ammonia occurs. In terms of the difference in the ground energy between subsystems, that for the adsorption on 3Ga-H is larger than that for the adsorption on N_ad_-H + Ga-H. This is the principal difference between the two adsorption systems and results in more ammonia adsorption on 3Ga-H than N_ad_-H + Ga-H. This is not because the probability flows towards lower energy eigenlevels but because the probability scatters to increase the entropy of the whole system.

### 3.2. Adsorption Probability

**Figure 3:**
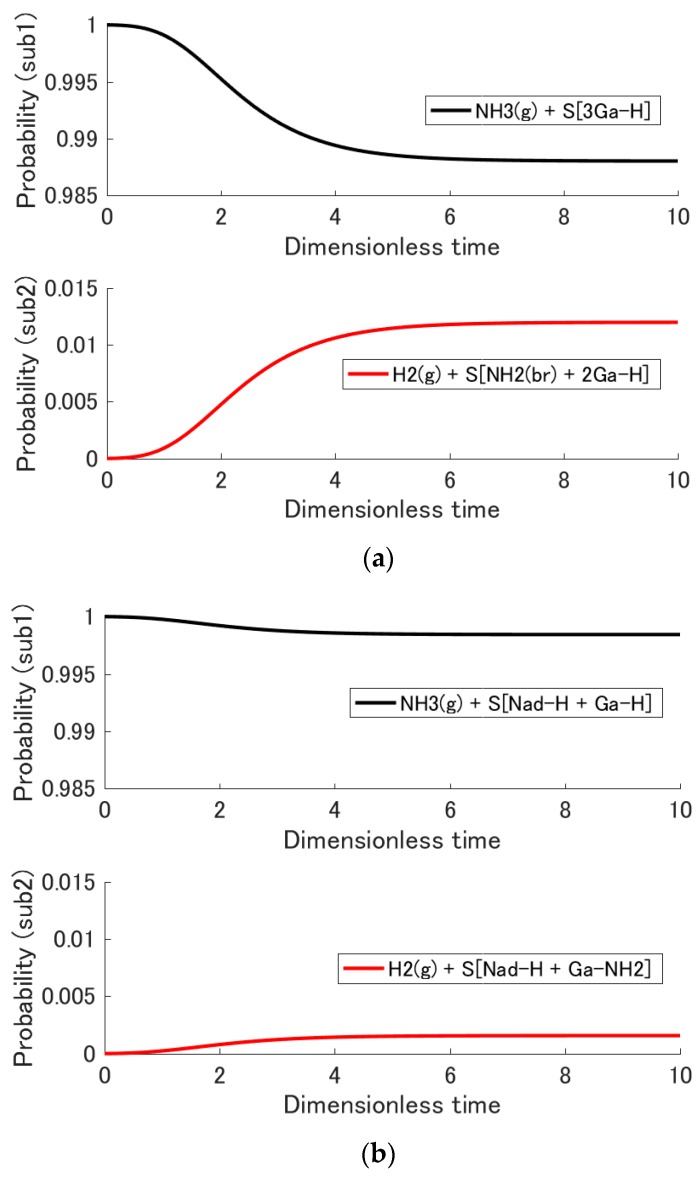
Evolution of the total probability of each subsystem as a function of the dimensionless time for the adsorption reactions on (**a**) S[3Ga-H] and (**b**) S[N_ad_-H + Ga-H]. This probability corresponds to the sum of the probabilities in Figure 2 over the energy eigenlevels.

Although the probability distribution $\left\{p_{i}\right\}$ is the raw information of state as shown in Section 3.1, in the case of hypoequilibrium state evolution, the two thermodynamic properties $P^{\mathrm{sub}}$ and $T^{\mathrm{sub}}$ provide additional useful state information as can be seen from Equation (15). The former, the total probability evolution of each subsystem, is discussed here, while the latter, the subsystem temperature, is discussed in Section 3.3. Figure 3a,b shows the subsystem probability evolution for each adsorption system. At the initial state, the total probability of subsystem 1 $P^{\mathrm{sub}1}$ is 0.99999 and the total probability of subsystem 2 $P^{\mathrm{sub}2}$ is 0.00001 as mentioned in Section 2.3. During state evolution, $P^{\mathrm{sub}1}$ decreases and $P^{\mathrm{sub}2}$ increases based on the principle of SEA. At the equilibrium state, $P^{\mathrm{sub}2}$ reaches 0.0120 and 0.0016 for the adsorption of NH_3_ on 3Ga-H and on N_ad_-H + Ga-H, respectively. In other words, ammonia is adsorbed on 3Ga-H approximately 7.5 times as much as on N_ad_-H + Ga-H. The sticking coefficient of ammonia on a GaN surface is reported in the literature to be 0.04 [49]; and it is this figure, which is used in GaN MOVPE models [50,51]. The value of $P^{\mathrm{sub}2}$ in the present study (i.e., 0.0120) is the same order of magnitude as the coefficient value found in the literature, although an exact comparison between these two properties cannot be made because the reconstructed surfaces in this paper are different from those in the literature.

**Figure 4:**
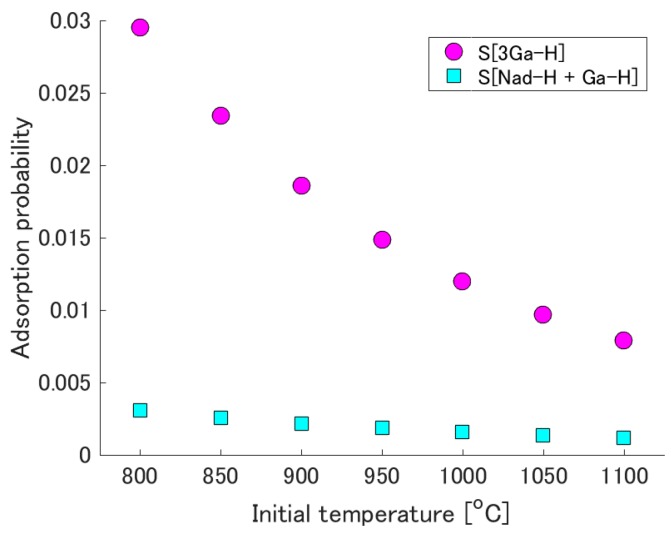
Initial temperature dependence of the adsorption probability at equilibrium.

To investigate the dependence of these equilibrium adsorption probabilities on initial temperature, the initial temperature is varied from 800 °C to 1100 °C. Figure 4 shows the equilibrium adsorption probability as a function of initial temperature. The decrease in this probability at higher initial temperatures is a reasonable tendency. The difference between the two equilibrium adsorption probabilities (i.e., that for each of the two reconstructed surfaces) becomes more significant at lower initial temperatures, and the difference at 800 °C is approximately one order of magnitude.

### 3.3. Temperature Increase by Adsorption

**Figure 5:**
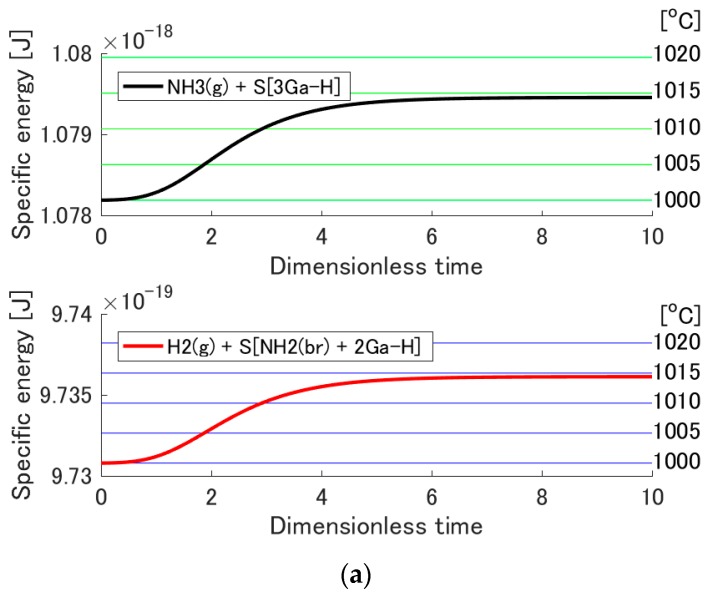

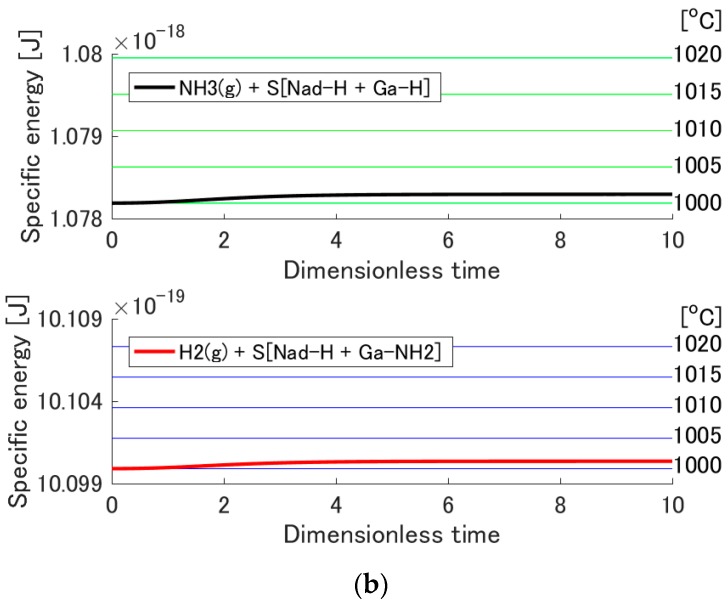
Evolution of the specific energy of each subsystem as a function of the dimensionless time for the adsorption reactions on (**a**) S[3Ga-H] and (**b**) S[N_ad_-H + Ga-H]. Green and blue horizontal lines correspond going from bottom to top to the specific energies at 1000, 1005, 1010, 1015, 1020 °C.

If the chemical adsorption occurs, the system temperature increases due to the released chemical energy. In general, one can see the probability distribution become wider and its peak shift towards the high-energy side as the temperature increases. However, it is difficult to observe that behavior in the present case (i.e., in Figure 2) since the temperature increase is not very large. To make it clearer, the evolution of the specific energy of each subsystem given by

(16) $$\frac{E^{\mathrm{sub}}}{P^{\mathrm{sub}}}=\frac{\sum p_{i}^{\mathrm{sub}}\epsilon_{i}^{\mathrm{sub}}}{\sum p_{i}^{\mathrm{sub}}},$$

is shown in Figure 5. As can be seen, this energy increases with system temperature. Figure 5 also shows the horizontal lines, which correspond to the specific energies at 1000, 1005, 1010, 1015, and 1020 °C (green lines for subsystem 1, blue lines for subsystem 2, and increasing temperature from bottom to top). By comparing the specific energy evolution of each subsystem with these horizontal lines, one can observe the temperature evolution of each subsystem. The temperature at equilibrium for adsorption onto 3Ga-H is estimated to be approximately 1015 °C because the black (or red) curve almost overlaps with the fourth green (or blue) line from below. The temperature at equilibrium for adsorption onto N_ad_-H + Ga-H is estimated to be approximately 1000 °C because of the position of the black (or red) curve relative to the first green (or blue) line from below. For adsorption onto N_ad_-H + Ga-H, the temperature increase is insignificant because the adsorption probability is quite small. However, for adsorption onto 3Ga-H, the temperature increase is much more important.

## 4. Conclusions

In this study, the non-equilibrium modeling of the chemical adsorption of ammonia onto GaN(0001) reconstructed surfaces under MOVPE conditions (3Ga-H and N_ad_-H + Ga-H on a 2 × 2 unit cell) is performed using the first-principle, non-equilibrium thermodynamic-ensemble based framework SEAQT. Results show that the adsorption probability on 3Ga-H is approximately 7.5 times higher than that on N_ad_-H + Ga-H for the case when the initial temperature is 1000 °C. This difference should affect the MOVPE process significantly. In addition, it is demonstrated that the difference in adsorption probability at equilibrium between the two reconstructed surfaces becomes much more significant the lower the initial temperature is.

Finally, the SEAQT framework is a powerful and useful theoretical tool for modeling non-equilibrium processes, particularly when a process starts from a highly non-equilibrium state such as that for chemical adsorption. The unique state evolution predicted is very helpful in clearly understanding the non-equilibrium process involved. In fact, use of the SEAQT framework should lead to better control of the MOVPE process through the selection of preferable reconstructed surfaces. Its wider use for other processes in which, for example, DFT is use to obtain information about the energetics at equilibrium, can as well provide useful information across multiple spatial and temporal scales about the kinetics of the process and its dynamics (i.e., provided τ is determined as described above) and can do so with a significantly smaller computational burden than that of conventional microscopic/mesoscopic approaches.
